# Postoperative Analgesic Effects of Different Doses of Epidural Hydromorphone Coadministered with Ropivacaine after Cesarean Section: A Randomized Controlled Trial

**DOI:** 10.1155/2019/9054538

**Published:** 2019-03-03

**Authors:** Meijuan Yang, Luyang Wang, Hong Chen, Yuwen Tang, Xinzhong Chen

**Affiliations:** Department of Anesthesiology, Women's Hospital, School of Medicine, Zhejiang University, No. 2 Xueshi Road, Hangzhou, Zhejiang 310006, China

## Abstract

**Purpose:**

Single dose of epidural hydromorphone has been introduced to serve as an alternative method for postcesarean section analgesia. However, optimal dose of epidural hydromorphone remains unknown. Hence, we evaluated and compared the analgesic and adverse effects of postoperative different doses of epidural hydromorphone coadministered with ropivacaine after cesarean section.

**Methods:**

Eighty term parturients with elective cesarean section under epidural anesthesia were allocated into four groups. Epidural analgesia was administered with an epidural bolus of either 0 mg (group H0), or 0.2 mg (group H1), or 0.4 mg (group H2), or 0.6 mg (group H3) hydromorphone coadministered with ropivacaine. The primary outcome was the visual analogue pain scores (VAPSs) and rescue opioid consumption (PCIA with sulfentanil) in 24 hours. Adverse effects such as respiratory depression, pruritus, nausea, and vomiting were recorded.

**Results:**

The VAPSs of group H1 at 2, 4, 6, 12 h and 24 h after surgery was similar to group H0. The VAPSs of group H2 at 4 and 6 h postoperatively were significantly decreased when compared to group H0. But, the VAPSs of group H2 at 2, 12, and 24 h postoperatively were similar to those of group H0. The VAPSs of group H3 at 4, 6, 12 h, and 24 h after surgery were significantly decreased when compared to those of group H0. The total sulfentanil consumption in 24 hours was 90 ± 26 *μ*g in group H0, 75 ± 29 *μ*g in group H1, 54 ± 32 *μ*g in group H2, and 15 ± 16 *μ*g in group H0. Adverse effects were comparable in the four groups.

**Conclusions:**

Epidural administration of 0.6 mg hydromorphone coadministered with ropivacaine after cesarean section provided satisfactory pain relief with less sulfentanil consumption. This trial is registered with ChiCTR-IPR-16010026.

## 1. Introduction

It is well known that the pain after cesarean section is usually ranked moderate to severe [1]. Adequate pain control after cesarean section with minimal adverse effects is important because women require a rapid recovery to ambulate and take care of their babies. Epidural analgesia generally provides superior postoperative pain relief compared to intravenous analgesia [2, 3]. Traditionally, epidural administration of opioids has been successfully used in bolus, continuous infusion, and patient-controlled epidural analgesia (PCEA) for pain control after cesarean section. A single dose of epidural morphine provides higher-quality analgesia compared with parenteral opioids [4], and it is commonly used due to its ease of application and low cost. But, the major drawbacks of morphine are its undesirable side effects, such as pruritus, nausea, and vomiting.

The hydrophilic properties of morphine make it ideal for long-acting analgesic. Owing to the high degree of hydrophilicity (octanol buffer distribution coefficient of 1), epidural morphine could provide highly effective analgesic with slow onset and longer duration but it is often accompanied by prolonged opioid side effects [5]. Hydromorphone, which was introduced into clinical practice in the 1920s, is not as extreme as morphine: it has moderate hydrophilicity (octanol buffer distribution coefficient of 525) [6]. Due to its hydrophilicity, epidural hydromorphone could cross the blood-brain barrier faster and provide fast onset and modest duration of action clinically [7, 8]. Neurxial hydromorphone had been shown to be as effective as morphine for postoperative analgesia in nonobstetric or obstetric patients with potentially lower incidence of adverse effect profile [9–11], so hydromorphone is a reasonable alternative to morphine for postcesarean section analgesia. Though there have been many postoperative analgesia studies with epidural hydromorphone, what remains to be determined is the appropriate dose. While several studies have determined the optimal dose for epidural morphine postoperatively [12], few similar studies exist for hydromorphone. In the present prospective and randomized study, we evaluated and compared the analgesic and adverse effects of postoperative epidural administration of different doses of hydromorphone coadministered with a fixed dose of ropivacaine after cesarean section.

## 2. Materials and Methods

The study protocol was approved by ethics committees of Women's Hospital, School of Medicine, Zhejiang University. 80 American Society of Anesthesiologists (ASA) I-II patients aged between 21 and 45 years scheduled for elective lower segment cesarean section were enrolled after signing their informed written consent. Exclusion criteria were patient's refusal, contraindication to epidural anesthesia (e.g., infection at the intended site of epidural needle insertion and neurologic defects such as transverse myelitis), severe pregnancy-induced hypertension, history of long-term opioid consumption, and history of allergy to any of the study medications.

Routine monitoring with 3-lead electrocardiogram (ECG), peripheral oxygen saturation (SpO_2_), and noninvasive blood pressure (NBP) was performed throughout the operation. Before anaesthesia, parturients were hydrated with lactated Ringer's solution 10 ml/kg for prehydration. Epidural anaesthesia was performed with the patient in the lateral decubitus position at the L1-2 or L2-3 interspace using the loss of resistance to saline technique with an epidural needle. After test dose of 3 ml 3% chloroprocaine, 3-4 cm of the closed end, a multiorifice epidural catheter was inserted into the epidural space and secured. A T4 sensory level to pinprick was achieved using 3% chloroprocaine, and then, surgery was started. If the mean arterial pressure decreases (>25% of baseline), ephedrine (5 to 10 mg intravenous) and Ringer's lactate solution were administered as necessary to maintain the NBP during the surgery. Tropisetron 5 mg was given intravenous intraoperatively for nausea and vomiting prophylaxis. At skin closure, an anesthetist, blinded to the drug, administered an epidural bolus containing different doses of hydromorphone plus 1.6 mg ropivacaine. All patients were assigned to four groups (H0, H1, H2, and H3); they received hydromorphone coadministered with a fixed dose of 1.6 mg ropivacaine via epidural injection. And, groups H0, H1, H2, and H3 received 0, 0.2, 0.4, and 0.6 mg hydromorphone, respectively. For all groups, the total volume injected into the epidural space was diluted with normal saline to 8 ml.

Patients who had inadequate anesthesia intraoperatively or received narcosis analgetics such as intravenous fentanil were excluded from data analysis. Before the patient was transferred to the postanesthesia care unit, the epidural catheter was removed and the patient-controlled intravenous analgesia (PCIA) was administered immediately using an electronic analgesia pump occupied with sulfentanil according to the following protocol: 0 *μ*g/h continuous dose, 2 *μ*g/h self-controlled dose, 10 min lock time, and 12 *μ*g/h controlled maximum dose. The dosage of sulfentanil is similar to previous studies [13]. All other NSAIDs, cycloxygenase-2 inhibitors, and opioids were prohibited during the postoperative period. Postoperative nausea or vomiting was treated with intravenous tropisetron as needed. For pruritus, nalbuphine 2.5 mg was administered intravenously as required. For respiratory depression (RR < 10/minute), naloxone 0.2 mg was administered intravenously.

All data collection was performed by a medical student who was unaware of group allocation and had not been involved in clinical care of the patients. The visual analogue pain scores (VAPSs) were explained to each patient in the operation room, and the patient's level of pain intensity was reviewed by the VAPSs on a 0–10 scale (a 10 cm linear scale, with 0 and 10 labeled as “no pain” and “worst pain imaginable”) during the 24 h postoperatively at 2 h, 4 h, 6 h, 12 h, and 24 h. The total sulfentanil consumption in 0–24 h period was calculated. Side effects like respiratory depression, pruritus, nausea, and vomiting were recorded. All antiemetic (tropisetron), antipruritic (nalbuphine), and naloxone consumption were recorded.

## 3. Statistical Analysis

Post hoc power analysis was performed at the end of the study, taking the 12 h VAPSs as the primary outcome measure. With 20 patients assigned to each group, the power of this study was 100% for the 12 h VAPSs with *α* equal to 0.05, so the sample size is enough in our study. Data were analyzed using SPSS 16.0 software (SPSS Inc., Chicago, IL, USA). Demographic and outcome data are summarized as mean ± SD or number as appropriate. Normal distribution was determined using the Kolmogorov-–Smirnov test. Differences between the four groups were compared using analysis of variance (continuous variable) for normally distributed variables, the Kruskal–Wallis test for nonparametric comparisons, and the chi-squared test for categorical variables. *P* value ≤ 0.05 was considered to be statistically significant.

## 4. Results

Eighty patients joined in the present study from December 2016 to June 2017, and no participant was excluded from the primary analysis. Surgical anesthesia using epidural was acceptable to all patients. There were no significant differences among the four treatment groups with regard to demographics (Table 1) and fluid infusion (data were not shown). None of the patients required additional analgesic during the intraoperative period.

The results for postoperative pain and sulfentanil used were shown in Table 2. The VAPSs of group H1 and the additional sulfentanil requirement in 0–24 h period after surgery were similar to group H0 (control group). The VAPSs of group H2 at 4 h and 6 h postoperatively and the total sulfentanil comsumption were significantly decreased when compared to group H0 (*P* < 0.05). The VAPSs of group H3 at 4, 6, 12 h, and 24 h after surgery and total sulfentanil used were significantly decreased when compared to group H0 (*P* < 0.05).

Table 3 shows the results of side effects. There was no significant difference in the incidence of nausea, vomiting, pruritus, and in the received antiemetics at the 24 h postoperatively between the groups. All the pruritus were mild in nature, and no patient required any treatment. There were no reported episodes of significant respiratory depression. No patient received naloxone.

## 5. Discussion

Single-dose epidural opioid can offer high-quality analgesia postoperatively, and it is commonly used without the need for an expensive pump. Epidural morphine is widely used to achieve postoperative pain control, but it still causes prolonged opioid side effects [14]. Some researchers try to find a substitute of morphine for postcesarean analgesia. Hydromorphone was selected as one of these analgesics; we assumed that adding hydromorphone to the epidural anaesthetic might provide satisfactory pain control during the first 24 h postoperative hours. In our present study during the first 2 h postoperatively, the VAPSs were low and similar in the four groups. In the 4 h and 6 h after surgery, both groups H2 and H3 showed lower VAPSs than group H0. In the 12 h and 24 h after surgery, only group H3 showed lower VAPSs than group H0. The patient in group H3 applied the lowest dose of sulfentanil in the four groups. Above these, we can conclude that 0.6 mg epidural hydromorphone provided satisfactory pain relief with reduced analgesic requirement in the first 24 h after surgery.

Epidural hydromorphone provides good pain relief and has side effects similar to morphine. To limit major and minor opioid side effects, the use of low-dose epidural opioids has been advocated. In a recent study, Marroquin et al. found that epidural injection of 0.6 mg hydromorphone provided good postoperative analgesia and only 25% of patients requested antipruritic medication [15]. In Chestnut's research, using epidural hydromorphone 1 mg, the rate of pruritus was up to 58% [16]. Therefore, we thought that epidural hydromorphone in a dose of 0.6 mg would be enough for managing acute postoperative pain after cesarean section but with unpredictable pruritus. Singh et al. showed that 1.5 mg epidural morphine provided noninferior postcesarean analgesia with fewer adverse effects compared with 3 mg epidural morphine [17]. In our hospital, we commonly use 1.5–2 mg of epidural morphine for the postcesarean analgesia. Because there are inadequate data concerning the equianalgesic ratio of epidural morphine to epidural hydromorphone, we chose the ratio 5 : 1–10 : 1 for parenteral morphine to parenteral hydromorphone [18, 19]. And considering that the side-effect profile appears better with lower doses of epidural hydromorphone, we chose to use 0.2 mg, 0.4 mg, and 0.6 mg epidural hydromorphone to seek the optimal dose of epidural hydromorphone for the pain control postoperatively.

Nausea and vomiting are commonly seen with epidural opioid administration. Shulman et al. found the incidence of nausea and vomiting was 9% with epidural hydromorphone [20]. Palmer et al. in a dose-response study examined different doses of epidural morphine (0, 1.25, 2.5, 3.75, or 5 mg), the quality of analgesia, and side effects in 60 parturients [21]. Quality of analgesia increased as the dose of epidural morphine was increased up to 3.75 mg; however, increasing the dose further to 5.0 mg did not enhance analgesia, and pruritus increased with the dose of epidural morphine. But, in our study, we did not find the epidural hydromorphone increased nausea in a dose-dependent manner; we found no statistically significant difference between the four groups. There were many factors influencing the occurrence of nausea and vomiting. Firstly, when the parturients did not gain good pain relief, they would use the PCIA. It was not surprising that we found no differences in the side-effect profiles as sufentanyl had similar adverse events. Secondly, in patients who undergo cesarean delivery with epidural anesthesia, these problems can be aggravated by uterine manipulation and peritoneal closure. For these reasons, it was necessary to use an antiemetic preventively. In our study, we used intravenous tropisetron 5 mg intraoperatively for nausea and vomiting prophylaxis.

Pruritus is a common and troublesome side effect of epidural opioid administration after cesarean section. Shulman et al. reported pruritus in one of the 21 patients who received single bolus of epidural hydromorphone (1.25–1.5 mg) [20]. Marroquin et al. found that pruritus occurred in 25% patients while adding 0.6 mg hydromorphone to epidural space [15]. In our present study, the occurrence of pruritus was low in all groups. Though the rate of pruritus is high in group H3, there is no statistically significant difference between groups. More researches should be carried out to find out what role hydromorphone plays in pruritus. Respiratory depression is the most worrisome complication and may occur within minutes or be delayed for hours after injection. Although respiratory depression has been reported after 1 mg of epidural hydromorphone [22], we found no patient with this feared side effect; all patients should be closely monitored in the first 24 h after surgery.

Several limitations existed in our present study. First, we did not clearly record the onset time and duration of pain relief with epidural hydromorphone. Second, the use of sulfentanil consumption as our primary outcome to measure analgesic efficacy may be modulated by the mother if she is worried that opioids may affect her or her baby. Third, we investigated just three doses of epidural hydromorphone for postoperative analgesia, if we had studied four or more larger different doses, the result may be different and more information related to side-effect profile of epidural hydromorphone would be known. Our next step is to study the median effective dose (ED50) of epidural hydromorphone for postoperative pain relief at 24 h and to identify what side effects are present at that dose.

## 6. Conclusion

Hydromorphone has been proposed as an alternative for morphine due to its similar lipophilicity (hydromorphone 1.11–1.35 vs. morphine 0.70–1.39) and analgesic efficacy [23]. Our study showed that 0.6 mg epidural hydromorphone could be an appropriate dose for the treatment of acute postoperative pain after cesarean section. This is a one-step study in determining a safe and effective dosage of epidural hydromorphone for postsurgical analgesia. Additional research is needed to compare both efficacy and side effects between hydromorphone and morphine.

## Figures and Tables

**Table 1:** Demographic and obstetric data.

	Group H0 (*n*=20)	Group H1 (*n*=20)	Group H2 (*n*=20)	Group H3 (*n*=20)
Age (years)	32 ± 4	32 ± 4	33 ± 3	33 ± 3
Weight (kg)	71 ± 6	69 ± 6	69 ± 8	70 ± 7
Height (cm)	162 ± 4	161 ± 3	160 ± 4	160 ± 4
BMI (kg/m^2^)	27 ± 2	27 ± 2	27 ± 2	27 ± 3
Gestation (weeks)	38 ± 1	38 ± 2	38 ± 1	38 ± 1
Operation time (min)	47 ± 9	45 ± 10	44 ± 13	41 ± 9

Data are mean ± SD. BMI = body mass index.

**Table 2:** Results for VAPSs at 2, 4, 6, 12, and 24 h after injecting epidural hydromorphone and total analgesic requirement (PCIA with sufentanil) in the first 24 h.

	Group H0 (*n*=20)	Group H1 (*n*=20)	Group H2 (*n*=20)	Group H3 (*n*=20)
2 h	0.9 ± 0.6	0.95 ± 0.5	0.65 ± 0.6	0.6 ± 0.5
4 h	2.8 ± 0.9	2.4 ± 0.7	1.3 ± 0.9^*∗*^	0.7 ± 0.6^*∗*^
6 h	2.8 ± 0.7	2.7 ± 0.7	2.1 ± 0.7^*∗*^	0.8 ± 0.7^*∗*^
12 h	3.1 ± 0.8	2.9 ± 0.7	2.8 ± 0.6	1.0 ± 0.9^*∗*^
24 h	3.2 ± 0.7	3.1 ± 0.6	3.2 ± 0.7	2.5 ± 0.8^*∗*^
Total sufentanil required at 24 h (*μ*g)	90 ± 26	75 ± 29	54 ± 32^*∗*^	15 ± 16^*∗*^

Data are mean ± SD. ^*∗*^*P* < 0.05 versus group H0. VAPSs = visual analogue pain scores. PCIA = patient-controlled intravenous analgesia.

**Table 3:** Side effects in the 24 hours after surgery.

	Group H0 (*n*=20)	Group H1 (*n*=20)	Group H2 (*n*=20)	Group H3 (*n*=20)
Nausea	5	5	4	4
Vomiting	2	1	1	1
Antiemetic used	0	0	0	1
Pruritus	0	0	1	4
Respiratory depression	0	0	0	0

Data are presented as the number of patients.

## Data Availability

The datasets used and analyzed during the current study are available from the corresponding author on reasonable request.

## Abbreviations

PCIA: Patient-controlled intravenous analgesia
VAS: Visual analogue scale
ECG: Electrocardiogram
SpO_2_: Peripheral oxygen saturation
NBP: Noninvasive blood pressure
BMI: Body mass index
ED50: Median effective dose.
